# The Application of Mobile Health in Self-Management Among Patients Undergoing Dialysis: Scoping Review

**DOI:** 10.2196/76880

**Published:** 2026-01-02

**Authors:** Qin Xu, Yulin Xu, Xiaoqin Liu, Xiaolin Ma

**Affiliations:** 1 Tongji Hospital, Tongji Medical College, Huazhong University of Science and Technology, No. 1095 Jiefang Avenue, Wuhan, Hubei, 430030, China, 86 15171430963

**Keywords:** kidney, self-management, mHealth, hemodialysis, peritoneal dialysis, mobile health

## Abstract

**Background:**

The incidence of end-stage renal disease continues to rise annually, with dialysis currently serving as the primary replacement therapy. The effectiveness of dialysis treatment and patients’ quality of life are highly dependent on their self-management. Mobile health (mHealth), which provides real-time medical support through portable devices, has become an essential tool for assisting patients undergoing dialysis in optimizing their self-management.

**Objective:**

This study aimed to systematically explore the core elements of self-management in patients undergoing dialysis and clarify the primary applications of mHealth, including types of mHealth, relevant theories and models, mHealth-based interventions, and evaluation indicators.

**Methods:**

This study was guided by Arksey and O’Malley’s methodology, PRISMA-ScR (Preferred Reporting Items for Systematic Reviews and Meta-Analyses Extension for Scoping Reviews), and PRISMA-S (Preferred Reporting Items for Systematic Reviews and Meta-Analyses Literature Search Extension). Databases, such as PubMed, Embase, CINAHL, PsycINFO, and Web of Science, were systematically searched from January 2010 until October 2025. The participants included in this study were patients undergoing dialysis, and the study design must incorporate quantitative research. Published protocols, reviews, editorials, conference papers, books, and non-English studies were excluded. The Mixed Methods Appraisal Tool was used to evaluate the quality of the included studies. Quantitative studies were extracted, mapped, and summarized. The results were collated and synthesized using a structured spreadsheet.

**Results:**

Out of 1483 relevant studies, this scoping review ultimately selected 34 studies involving 2068 patients undergoing dialysis. Self-management among patients undergoing dialysis in this study included 6 major areas, including self-monitoring, diet and fluid management, medication management, disease-related knowledge, exercise management, and psychological management. Most studies used a single app (n=22) for management of patients undergoing dialysis, followed by 2 or more online interventions (n=6) and a remote patient monitoring system (n=3). The mHealth-based interventions in this study focused on self-monitoring, dietary and fluid management, and medication management. The transtheoretical model and stages of change (n=5), self-efficacy theory (n=4), and social cognitive theory (n=4) were the most commonly used theories. Among the evaluation indicators, interdialytic weight gain (n=12), serum potassium (n=14), serum phosphorus (n=20), and serum albumin (n=14) were the most commonly used objective indicators. Subjective indicators were assessed using scales, primarily covering adherence (n=17), self-efficacy (n=14), quality of life (n=12), knowledge (n=9), and diet and nutrition (n=9).

**Conclusions:**

Although mHealth holds promise for improving self-management and outcomes among patients undergoing dialysis, there remains significant room for advancement. Future research in this field should focus on enhancing adaptive software development, deeply integrating artificial intelligence technologies, addressing the needs of special populations, and establishing a standardized self-management evaluation system. Our findings not only provide a theoretical framework for optimizing clinical management strategies for patients undergoing dialysis but also offer targeted guidance and practical insights for the subsequent development of apps.

## Introduction

End-stage renal disease (ESRD) refers to the end stage of various chronic kidney diseases (CKDs). The global prevalence of patients with renal failure receiving dialysis treatment continues to rise, with the latest estimate reaching 823 per million population [1,2]. Although kidney transplantation is the treatment of choice for patients with ESRD, the majority of patients still rely on dialysis due to the shortage of donor kidneys [3]. Hemodialysis and peritoneal dialysis (PD) are the 2 most common types of dialysis. Although hemodialysis and PD have significantly improved survival rates among patients with ESRD [1,4], the invasive and long-term treatments also substantially increase the risk of dialysis-related complications or infections. Studies have shown that comorbidities, such as hypertension, diabetes mellitus, hyperkalemia, and hyperphosphatemia, and cardiovascular diseases are common in patients undergoing dialysis [1,5]. The quality of life and survival rate of patients undergoing dialysis also decline with increasing dialysis duration [5,6]. The quality of life and survival outcomes of patients undergoing dialysis are closely related to the quality of dialysis treatment, which in turn is directly dependent on the level of the patient’s self-management [7].

Self-management encompasses multiple aspects of health management. According to Lorig et al [8], self-management involved medical management (special dietary adherence and medication adherence), role management, and emotion management. In patients undergoing dialysis, self-management refers to whether the patients perform self-monitoring, strict control of diet (sodium, potassium, phosphorus, and other micronutrients) and fluid intake, regular medication administration, and prevention and management of complications. Patients undergoing dialysis can reduce negative symptoms and improve the quality of life through self-management behaviors [7].

Disease management of patients undergoing dialysis is a difficult point in the current medical work. The results of several studies have shown that the overall self-management level of patients undergoing dialysis was low [9,10]. Patients undergoing dialysis generally lacked knowledge of the disease, and their self-management behaviors, such as dietary control, fluid intake, and treatment adherence, fell short of standards [10]. The proposal of mobile health (mHealth) provides new ideas and methods for the remote management of patients undergoing dialysis. mHealth is a medical and public health service initiative based on mobile communications technology delivered through mobile phones, monitoring devices, personal digital assistant devices, and other wireless devices [11]. Because of its unique convenience, it has significant advantages in monitoring diseases, controlling symptoms, and promoting healthy behaviors.

There is a growing body of research on the role of mHealth in improving self-management in patients undergoing dialysis [12-15]. Most studies concentrate on developing mobile apps specifically designed for patients undergoing dialysis, that is, specialized software tools running on mobile devices. Additionally, telephones and SMS text messaging are also commonly used tools. Despite the growing interest in mHealth, evidence on mHealth-based self-management among patients undergoing dialysis remains limited. Therefore, this review aims to provide an overview of the use of mHealth in the self-management of patients undergoing dialysis, examine existing interventions, and summarize existing evaluation tools. The goal is to empower patients undergoing dialysis through mHealth, improve their self-management to enhance prognosis, and provide a practical reference for subsequent app development.

## Methods

### Overview

A scoping review based on the 5-stage methodological framework of Arksey and O’Malley [16], involving (1) identifying the research question, (2) identifying relevant studies, (3) study selection, (4) charting the data, and (5) collating, summarizing, and reporting the results [16]. The PRISMA-ScR (Preferred Reporting Items for Systematic Reviews and Meta-Analyses Extension for Scoping Reviews) [17] and PRISMA-S (Preferred Reporting Items for Systematic Reviews and Meta-Analyses Literature Search Extension) guidelines [18] (Checklist 1) were used as the protocol for this study. The study quality assessment was conducted using the 2018 version of the Mixed Methods Appraisal Tool, a tool specifically designed to evaluate the quality of qualitative, quantitative, and mixed methods studies [19].

### Stage 1: Identifying the Research Questions

The study population was adult patients on dialysis (both hemodialysis and PD), and the type of intervention was a service using mHealth. The primary objective of this review was to explore the use of mHealth in the self-management of patients undergoing dialysis. The research questions were based on an initial literature search and were refined during discussions in the research team.

In accordance with the overall objectives of this review, we have refined the research questions as follows:

1. What are the types of mHealth in the included studies?
2. What are the attributes of self-management in the included studies?
3. What are the interventions for mHealth-based self-management?
4. What are the evaluation tools for self-management in the included studies?

### Stage 2: Identifying Relevant Studies

A systematic literature search was conducted in PubMed (National Center for Biotechnology Information), Embase (Ovid), CINAHL (EBSCOhost), PsycINFO (EBSCOhost), and Web of Science (Clarivate Analytics) to identify studies relevant to the research objectives. We limited the search to studies that were published from January 2010 to October 2025. First, we identified the search strategy and search terms through group discussion. Then, a presearch was conducted in the database using the search strategy and search terms, and the search strategy and search terms were adjusted according to the search results. Subsequently, formal searches were conducted in 5 databases using the identified search strategies and search terms. Two keywords “mHealth” and “dialysis” were used in combination to cover the 2 main concepts of the research question. The specific search strategy and updated search methods can be found in Multimedia Appendix 1. The reference lists of all eligible studies were examined to identify any potentially relevant studies.

### Stage 3: Study Selection

Included studies were required to fulfill the following criteria. (1) Participants: adult patients (age≥18 years) receiving long-term dialysis with no gender restrictions; (2) Concept: studies were included if they addressed mHealth and self-management. Self-management is the focus of this scoping review. Among patients undergoing dialysis, we defined self-management as knowledge, diet and fluids, dialysis treatments, medications, dialysis access, exercise, and psychology. mHealth refers to health and medical services (including remote monitoring, health education, online counseling, etc) that are delivered using mobile devices (eg, smartphones and tablets); (3) Context: dialysis occurs at home or in a hospital. Eligible study designs must include quantitative research. We excluded published research protocols, reviews, editorials, conference papers, books, and non-English studies. Finally, if the full text cannot be obtained, the study will be excluded.

EndNote (Clarivate Analytics) software was used to identify duplicates and manage literature. The literature was screened by 2 trained reviewers (QX and YX). In the first stage, 2 reviewers independently reviewed the titles and abstracts of studies based on inclusion and exclusion criteria. Then, the 2 reviewers continued to independently screen the full text. In the second stage, 2 reviewers assessed the quality of the included literature based on the Mixed Methods Appraisal Tool. When 2 reviewers disagree, a third reviewer (Xiaoqin Liu) will join the discussion until all reviewers reach consensus, ensuring the rigor of the selection process.

### Stage 4: Charting the Data

The research team worked together to develop a data chart to guide the extraction of key information from each study. Descriptive chart information includes (1) a general description of the study, such as first author and year, country, study design, patient population, and purpose of the study; and (2) intervention-specific information, including type of mHealth, primary function, method of implementation, intervention time, and evaluation tools.

### Stage 5: Collating, Summarizing, and Reporting the Results

The research team summarized the data iteratively. Descriptive analyses were used to summarize the types of mHealth and self-management evaluation tools, while thematic content analyses were used to summarize the attributes of self-management and the content of mHealth-based self-management. First, codes were developed and applied to analyze the data. Coded segments of the data chart were then created with color-coded quotations, and the coding results were summarized in an Excel (Microsoft Corp) sheet. The Excel sheet was sorted by code and density. Key themes were extracted by analyzing the studies in an overall iterative comparison.

### Ethical Considerations

Ethical approval was not needed for this review.

## Results

### Basic Characteristics of the Included Studies

**Figure 1.**
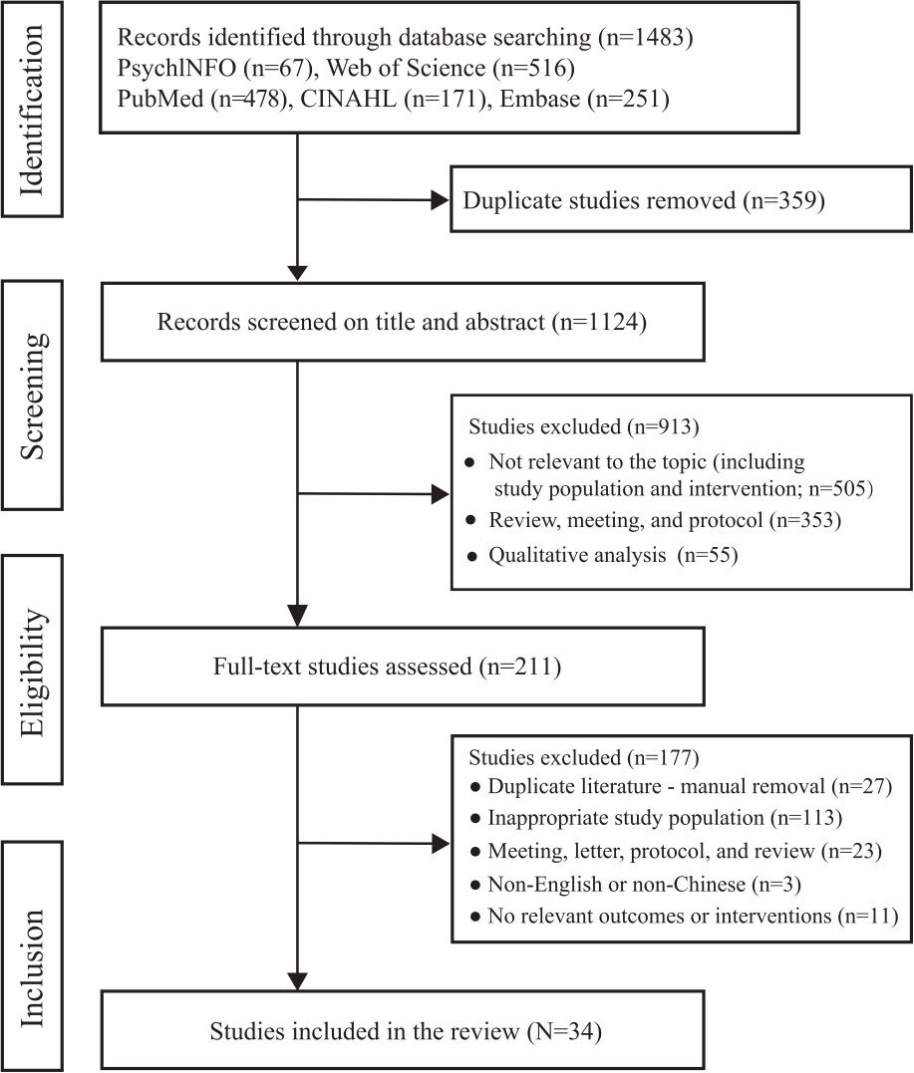
Flowchart outlining the search process for studies across databases, following the PRISMA-ScR (Preferred Reporting Items for Systematic Reviews and Meta-Analyses Extension for Scoping Reviews) guidelines.

We retrieved 1483 records from PsychlNFO (n=67), Web of Science (n=516), PubMed (n=478), CINAHL (n=171), and Embase (n=251). In total, 359 studies were excluded due to duplication, and 913 studies were excluded after reading the title and abstract. There were still 211 studies that needed to be read in full. After reading the full text, a total of 34 studies [12-15,20-49] were included in this scoping review (Figure 1).

**Figure 2.**
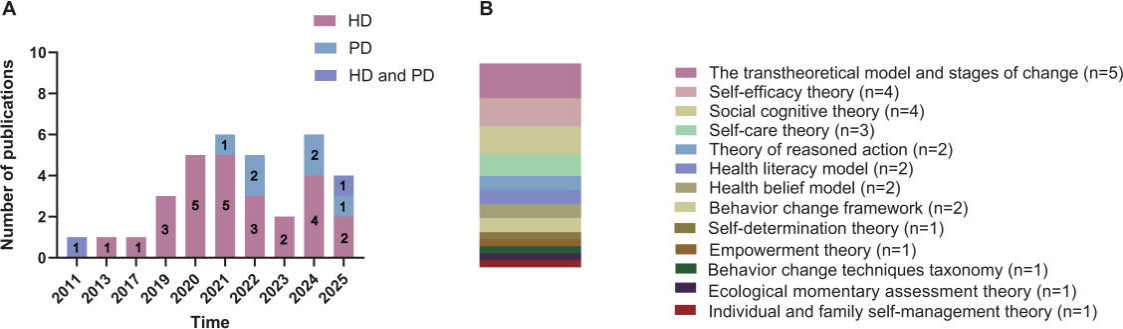
(A) The trend analysis of publication dates in the included studies. (B) The specific information on theories and models used to guide the content of mHealth self-management interventions. HD: hemodialysis; mHealth: mobile health; PD: peritoneal dialysis.

A total of 34 studies [12-15,20-49] were included in this study, involving 2068 patients undergoing dialysis. Among the included studies, 26 studies [12,13,20-26,28-44] involved patients undergoing hemodialysis, 6 studies [14,15,27,45-47] focused on patients undergoing PD, and 2 studies [48,49] encompassed patients undergoing both hemodialysis and PD. In terms of the regional distribution of the included studies, most of the studies were concentrated in Asia and North America. Among the 34 studies, Korea was dominated with 8 (23.53%) studies [13,15,23,27-29,35,46], followed by 5 (14.71%) studies [25,38,40,44,48] in the United States, 3 (8.82%) studies [12,24,47] in China, 3 (8.82%) studies [21,31,39] in Iran, 3 (8.82%) studies [26,30,49] in Australia, and 3 (8.82%) studies [14,37,42] in Thailand. Around 2 (5.88%) studies [20,22] in the Netherlands, 2 (5.88%) studies [32,45] in Japan, and 2 (5.88%) studies [34,43] in Indonesia each contributed to 2 studies, while 1 (2.94%) study [41] in Malaysia, 1 (2.94%) study [33] in Turkey, and 1 (2.94%) study [36] in Brazil each contributed 1 study. The studies were published mainly between 2019 and 2025, with a total of 31 studies [12-15,20-24,26-31,33-47,49]. Three studies were published in 2011, 2013, and 2017 (Figure 2A). The results of the quality assessment indicated that most studies demonstrated good quality. Specific details of the quality assessment can be found in Multimedia Appendix 2. Table 1 provides an overview of the key characteristics of the included studies. Additionally, Multimedia Appendix 3 presents the intervention type, core intervention contents, and evaluation indicators for each study.

**Table 1.** Summary of basic characteristics of the included studies.

Author and year	Country	Aim of the study	Study design	Population	Total sample (experimental/control group)
Saadatifar et al [39], 2022	Iran	To explore the impact of mHealth^a^ on treatment adherence in patients undergoing HD^b^.	Quasi-experimental study	HD	80 (40/40)
Ren et al [24], 2019	China	To explore the impact of a WeChat-based health education program on the self-management of patients undergoing HD.	A longitudinal experimental intervention study	HD	85 (49/36)
Park and Kim [23], 2019	South Korea	To evaluate the impact of a program based on app and SMS text messaging for patients undergoing HD.	Quasi-experimental study	HD	84 (42/42)
Pack and Lee [35], 2021	South Korea	To develop a mobile app for dietary management and evaluate the impact on patients undergoing HD.	RCT^c^	HD	75 (37/38)
Fakih El Khoury et al [22], 2020	Netherlands	To evaluate the efficacy of the app-based dietary intervention for patients undergoing HD.	A pilot study: a before-and-after study	HD	23
Hanifi et al [31], 2019	Iran	To assess the impact of counseling and follow-up phone calls on patients undergoing HD.	Quasi-experimental study	HD	86 (43/43)
Cho and Park [28], 2020	South Korea	To assess the impact of a tablet-based self-management program on patients undergoing HD.	Quasi-experimental study	HD	46 (23/23)
Chiang et al [12], 2021	China	To evaluate whether the Assisted Care Program in the app can help patients better control their dietary phosphorus intake.	Quasi-experimental study	HD	60 (30/30)
Zwi et al [26], 2022	Australia	To evaluate the feasibility of the app and its impact on patients undergoing HD.	A mixed methods study	HD	61
Welch et al [25], 2013	United States	To evaluate the impact of mobile programs on diet and fluid intake in patients undergoing HD.	A pilot study	HD	33 (16/17)
Thongsunti et al [42], 2024	Thailand	To evaluate the effectiveness of telemedicine-based management of hyperphosphatemia in patients undergoing HD.	RCT	HD	80 (40/40)
Chung et al [29], 2024	South Korea	To assess the impact of adaptive nutrition and education on patients undergoing HD using real electronic medical record data.	A decentralized clinical trial	HD	153 (42/40/34/37)^d^
Dawson et al [30], 2021	Australia	To assess the impact of SMS text messaging on patients undergoing HD.	A randomized feasibility study	HD	115 (78/37)
Fakih El Khoury et al [20], 2021	Netherlands	To assess the efficacy of an intervention using the app on phosphorus.	A pilot study: a before-and-after study	HD	23
Hosseini et al [21], 2023	Iran	To determine the effect of an app on self-efficacy and self-care of patients undergoing HD.	Quasi-experimental study (longitudinal single-group study)	HD	54
Min and Park [13], 2020	South Korea	To assess the impact of a mobile app–based self-management support program on older adults undergoing HD.	Quasi-experimental study	HD	56 (28/28)
Mollaoğlu et al [33], 2024	Turkey	To evaluate the impact of education and art therapy through a telemedicine approach in patients undergoing HD.	RCT	HD	60 (30/30)
Rocco et al [38], 2023	United States	To evaluate the impact of an app on self-monitoring of daily fluids in patients undergoing HD.	A pilot study: a before-and-after study	HD	18
St-Jules et al [40], 2021	United States	To assess the feasibility and acceptability of mHealth for managing hyperphosphatemia in patients undergoing HD.	A feasibility trial	HD	40 (13/14/13)^e^
Teong et al [41], 2022	Malaysia	To evaluate the effectiveness of an app for nutritional management in patients undergoing HD.	RCT	HD	66 (33/33)
Pungchompoo et al [37], 2024	Thailand	To evaluate the impact of a home telemedicine model on older patients undergoing HD.	A mixed methods study	HD	54 (24/30)
Nursalam et al [34], 2020	Indonesia	To evaluate the impact of the app on improving fluid restriction adherence in patients undergoing HD.	A mixed methods study	HD	60 (30/30)
Hayashil et al [32], 2017	Japan	To evaluate the usefulness of the self-management support system for self-monitoring in patients undergoing HD.	A pilot study	HD	18 (8/10)
Pinto et al [36], 2020	Brazil	To evaluate the impact of the app on fluid restriction and dietary control in patients undergoing HD.	A randomized, single-center, self-controlled study	HD	48
Andriati [43], 2025	Indonesia	To evaluate the impact of the app on adherence and renal function of patients undergoing HD.	Quasi-experimental study	HD	55
Taguiam [44], 2025	United States	To evaluate the impact of the app on fluid intake management and body weight in patients undergoing HD.	A mixed methods study	HD	18
Lee and Kang [15], 2024	South Korea	Using a mobile instant messaging tool to customize diet plans for patients undergoing PD^f^ and evaluate outcomes.	Quasi-experimental study	PD	43 (21/22)
Chae and Kim [27], 2024	South Korea	To develop the app for improved self-management and evaluate its impact on patients undergoing PD.	RCT	PD	53 (27/26)
Uchiyama et al [45], 2022	Japan	To assess the impact of using a remote patient monitoring system on patients undergoing PD.	A randomized crossover controlled trial	PD	15
Jung et al [46], 2021	South Korea	To evaluate the impact of remote patient monitoring on automated patients undergoing PD.	RCT	PD	50 (28/22)
Lukkanalikitkul et al [14], 2022	Thailand	To evaluate the availability and impact on patients undergoing PD for app.	User-centered design study	PD	9
Zeng et al [47], 2025	China	To evaluate the effectiveness of a PD management system in improving adherence and clinical outcomes.	A retrospective cohort study	PD	127
Stark et al [48], 2011	United States	To assess the effectiveness of a PDA^g^-based app for dietary management in patients undergoing dialysis.	RCT	PD, HD	HD:19 (9/10); PD: 21 (11/10)
Beer et al [49], 2025	Australia	To evaluate the effectiveness of the app in controlling serum phosphorus levels in patients undergoing dialysis.	RCT	PD, HD	180 (90/90)

a mHealth: mobile health.

b HD: hemodialysis.

c RCT: randomized controlled trial.

d The study divided participants into four groups: (1) control (n=42), (2) education intervention (n=40), (3) meal intervention (n=34), and (4) education and meal interventions (n=37).

e The grouping is set up as follows: (1) educational videos and handouts (Education; n=13), (2) education intervention plus mobile self-monitoring with email feedback (Monitoring; n=14), or (3) education and monitoring interventions plus social cognitive theory-based behavioral videos (Combined; n=13).

f PD: peritoneal dialysis.

g PDA: personal digital assistant.

### Theories or Models Involved

Among the studies included, 14 [14,15,29,31-33,36,37,39,41,43,45-47] did not explicitly mention the theories or models involved. Of these studies, 5 [14,15,45-47] were in patients undergoing PD, and 9 [29,31-33,36,37,39,41,43] involved patients undergoing hemodialysis. Nine [13,20-27] studies combined 2 or more theories or models, and 11 [12,28,30,34,35,38,40,42,44,48,49] studies involved only 1 theory or model.

Among mHealth-based self-management interventions for patients undergoing dialysis, the most frequently incorporated theories included the transtheoretical model (TTM) and stages of change (n=5 [20,22,24,26,42]), self-efficacy theory (n=4 [12,21,24,35]), social cognitive theory (n=4 [25,27,40,48]), and Orem’s theory of self-care (n=3 [21,23,28]). Most studies have focused their attention on theories related to behavior change (such as the TTM, the theory of reasoned action [20,22], the health belief model [13,34], behavior change frameworks [30,49], and the behavioral change techniques taxonomy [38]). Additionally, Bandura self-efficacy theory and Oren self-care theory were often used in combination with other theories. Accordingly, we visualized the theories or models involved in this study (Figure 2B).

### Attributes of Self-Management in Patients Undergoing Dialysis

**Figure 3.**
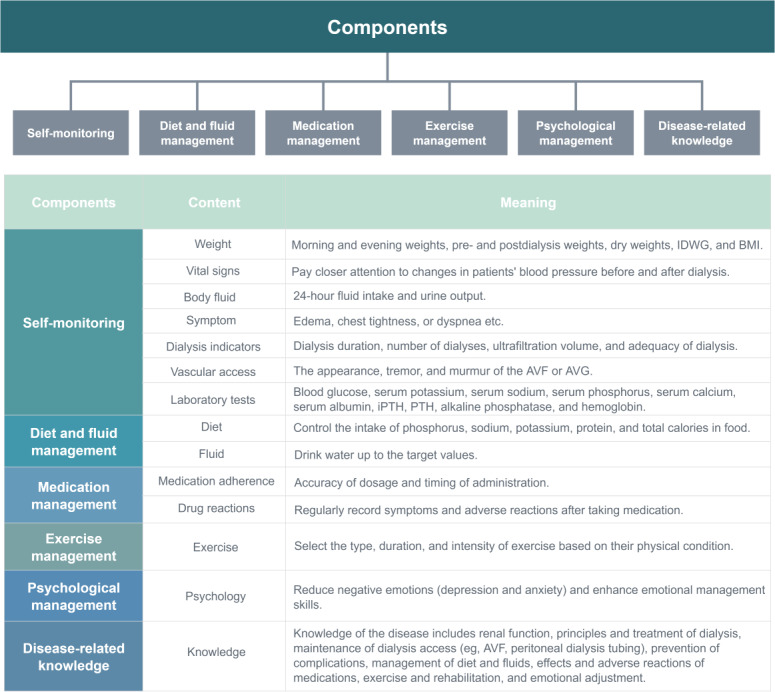
Components of self-management for patients undergoing dialysis. AVF: arteriovenous fistula; AVG: arteriovenous graft; IDWG: intradialytic weight gain; iPTH: intact parathyroid hormone; PTH: parathyroid hormone.

We categorized the self-management included in this study into six main themes, which were (1) self-monitoring, (2) diet and fluid management, (3) medication management, (4) exercise management, (5) psychological management, and (6) disease-related knowledge. The components of self-management for patients undergoing dialysis and their corresponding explanations are detailed in Figure 3.

### mHealth-Based Self-Management Intervention Program

The mHealth-based self-management intervention program is focused on the type of mHealth, the content of the mHealth-based intervention, and the duration of the intervention.

The types of mHealth mentioned in this study included app (based on mobile device, tablet personal computer, or personal digital assistant), a remote patient monitoring system, SMS text messaging, and telephones. Apps were divided into 2 categories, such as dialysis-specific software and instant messaging software (eg, WeChat [Tencent Holdings Limited], Line [LY Corporation], Facebook [Meta Platforms, Inc], WhatsApp [Meta Platforms, Inc], and KakaoTalk [Kakao Corp]). Two studies [29,30] intervened via SMS text messaging only, 1 study [31] via telephone only, 4 studies [15,24,33,42] via instant messaging software only, and 18 studies [12-14,20-22,25,26,28,34-36,38,41,43,44,47,49] via dialysis-specific software only. Six studies [23,27,37,39,40,48] used 2 or more online interventions. There were only 3 studies [32,45,46] based on a remote patient monitoring system, 1 [32] for patients undergoing hemodialysis and the remaining 2 [45,46] for patients undergoing PD.

**Figure 4.**
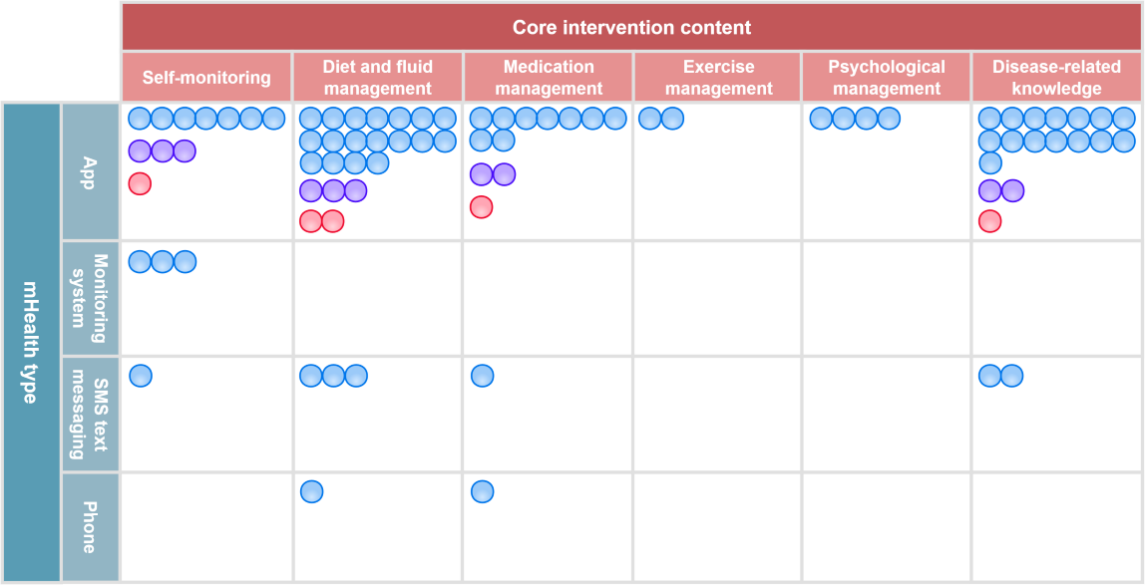
Gap map of core self-management interventions across different mobile health (mHealth) categories for patients undergoing dialysis.

This scope review categorized the app’s content into the following dimensions, including intelligent education hub, full-dimensional monitoring system, accurate nutritional management, behavioral interventions (fluid, exercise, and medication adherence), intelligent reminders and alerts, doctor-patient collaboration network, and social support system. Remote patient monitoring systems placed more emphasis on remote monitoring, alarms, and dynamic interventions for patients undergoing dialysis. The details could be found in Table 2. Interventions delivered via apps and remote patient monitoring systems were more comprehensive and satisfactory than those delivered via phone and SMS text messaging, even though they showed similar functionalities in certain aspects. Figure 4 illustrates the gap map of core self-management interventions across different mHealth categories for patients undergoing dialysis (blue circles indicate studies with hemodialysis as the research participants; purple circles indicate studies with peritoneal dialysis as the research participants; red circles indicate studies with hemodialysis and peritoneal dialysis as research participants).

**Table 2.** Type of mobile health and mobile health–based interventions to improve self-management in patients undergoing dialysis.

Classification of mHealth^a^	Numbers	Function and intervention contents	
App			
Dialysis-specific software	24	Intelligent education hub Disease knowledge base: kidney function, dialysis principles, complication prevention, medications, and lifestyle guidance presented through animated videos, podcasts, manuals, and charts Operating system training: training for new patients (including equipment operation instruction) and periodic knowledge reinforcement. Full-dimensional monitoring system Automatic acquisition and manual entry of data: physiological indicators, dialysis parameters, symptom logs, and medication records Visualization analysis: trend analysis and correlation analysis. Accurate nutritional management Database support: 500+kinds of kidney disease-specific food nutrients, nutrition calculator (real-time display of phosphorus, sodium, potassium, and protein) Recording function: barcode scanning to enter food, manual recording of intake Analysis and feedback: nutritional value calculation for each meal, health scoring system (weekly or monthly summary), electrolyte excess warning (sodium, potassium, and phosphorus) Behavioral interventions: personalized dynamic recipe recommendations (adjusted based on lab data), daily water intake limits (residual urine volume algorithm) Behavioral interventions (fluid, exercise, and medication adherence) Personalized goal management: goal setting, progress tracking (instant values + trend charts + health scores) Behavior shaping tools: badge reward mechanism (continuous recording of achievements) Intelligent reminders and alerts Treatment reminder: dialysis time and follow-up appointment Medication reminder: medication time, dosage, and drug interaction Early warning system: set thresholds to trigger abnormal alerts and notify patients and health care at the same time. Doctor-patient collaboration network Prescription cloud adjustment, test result synchronization (direct connection to electronic medical records), and equipment interconnection. Social support system Patient community: experience sharing (recipes or exercise programs) Family linkage: caregiver collaborative recording function and family health data sharing. System Infrastructure System compatibility: mobile (iOS/Android)+Web+PDA^b^ compatibility, offline data caching, and synchronization Intelligent devices: support joint NFC^c^, OCR^d^, and PDA terminals Multimodal recording: support voice recording, image recognition, and manual supplementation.	
Instant messaging software	5	Knowledge delivery and personalized guidance: basic health manuals, video educational resources, and customized content delivery Dietary interventions and guidance Psychological intervention: structured facilitation (motivational interviewing), psychological facilitation (drawing healing-experts’ video guidance), and emotional support (full psychological status tracking) Instant interaction: online Q&A^e^ with health care, regular phone consultations (psychological support and health guidance), and videoconference support (group discussions or personalized guidance).	
A remote patient monitoring system	3	Intelligent monitoring hub Device interconnection: bidirectional communication with automatic peritoneal dialysis devices through a cloud platform Full-dimensional data collection: real-time access to dialysis parameters, device alarm logs, and patient physiological indicators. Remote dynamic intervention Intelligent alarms: yellow and red alarms, instantly triggering phone interventions; Prescription cloud adjustment: physicians remotely optimize automated peritoneal dialysis prescription parameters based on real-time data Physician-patient collaboration platform: support real-time treatment issues through system messages and phone calls.	
Telephones	4	Health education Treatment adherence assessment Personalized dietary recommendations Psychological support Problem solving	
SMS text messaging	4	Personalized health education Regular collection of patients’ feedback Regular medication reminders Positive motivational text messages	

a mHealth: mobile health.

b PDA: personal digital assistant.

c NFC: near field communication.

d OCR: optical character recognition.

e Q&A: question-and-answer.

The duration of the intervention ranged from 2 to 24 weeks. The included studies preferred interventions for 3 months [12,24,36,39,43,49], 6 months [21,30,37,40], 12 weeks [26,31,33,41], 2 weeks [20,22,32], 8 weeks [23,29,35].

### Key Indicators and Evaluation Tools on Self-Management

**Figure 5.**
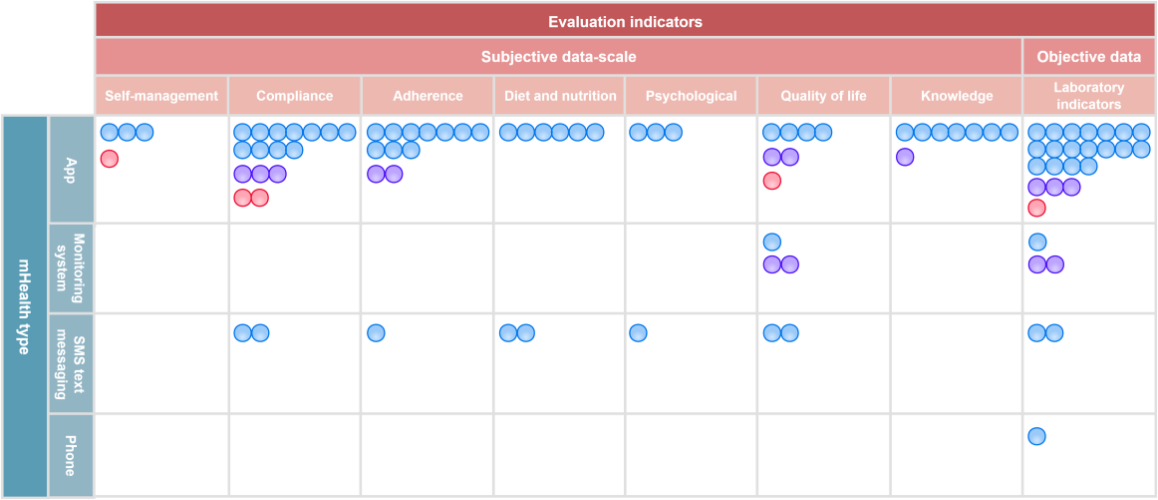
Gap map of self-management assessment indicators across different mobile health (mHealth) categories for patients undergoing dialysis.

Indicators for assessing self-management in patients undergoing dialysis were divided into 2 categories, namely subjective and objective indicators. The subjective indicators mainly involved scales, such as measuring self-management, adherence, self-efficacy, literacy, depression, anxiety, perceived benefits, and quality of life of patients undergoing dialysis. Objective indicators included weight, blood pressure, and laboratory tests. Of these, intradialytic weight gain, serum albumin, serum potassium, and serum phosphorus were the most mentioned indicators in the included studies. Detailed information is provided in Table 3. Figure 5 illustrates the gap map of self-management assessment indicators across different mHealth categories for patients undergoing dialysis (blue circles indicate studies with hemodialysis as the research participants; purple circles indicate studies with peritoneal dialysis as the research participants; red circles indicate studies with hemodialysis and peritoneal dialysis as research participants).

**Table 3.** Mobile health–based indicators for assessing self-management in patients undergoing dialysis.

Items	Numbers	Tools
Self-management	4	The self-management scale for patients undergoing hemodialysis [24,50] The self-care performance questionnaire [21] The Perceived Medical Condition Self-Management Scale (PKDSMS) [51] The Patient Activation Measure-13 (PAM-13) [52]
Adherence	17	The End-Stage Renal Disease Adherence Questionnaire (ESRD-AQ) [53] Hemodialysis Compliance Questionnaire [43] The Simplified Medication Adherence Questionnaire (SMAQ) [54] The Dialysis Diet and Fluid Nonadherence Questionnaire (DDFQ) [55] The Modified Morisky Scale (MMS) The Compliance of Patient Role Behavior Tool [23] The Sick-role Behavior Adherence [13] The Adherence Questionnaire (self-developed or revised version)
Self-efficacy	14	The 15-item dietary self-efficacy questionnaire [56] The decision self-efficacy scale [57] The 11-item Fluid Self-Efficacy Scale (FSES) [25] The 6-item Chronic Disease Self-Efficacy Scale (CDSES) [24] The Self-Efficacy for Appropriate Medication Use Scale (SEAMS) [58] The strategies used by people to promote health The Self-Efficacy Scale (self-developed or revised version) [59]
Perceived benefits	2	The Benefits of Sodium Adherence (BSA) [25,60] The 9-item Benefits of Fluid Adherence Scale [25,61]
Literacy	2	The Media Health Literacy Questionnaire (MeHLit) [62] The Health Literacy Questionnaire (HLQ) [63]
Knowledge	9	Self-developed or revised knowledge-related questionnaires
Quality of life	12	Kidney Disease Quality of Life Instrument-Short Form (KDQOL-SF) [64] The Kidney Disease Quality of Life (KDQOL-36) [65] Short Form 36 (SF-36) EuroQol Five Dimensions Questionnaire (EQ-5D) Health-Related Quality of Life (HRQoL) 9-item Thai Health Status Assessment Instrument (9-THAI) [66]
Diet and nutrition	9	24-hour dietary recall method 3-day dietary recall method App automatic calculation Food Frequency Questionnaires (FFQ) Malnutrition Inflammation Score (MIS) Healthy Eating Index (HEI-20)
Psychosocial	3	Beck Depression Inventory (BDI) Beck Anxiety Inventory (BAI)
Weight, dry weight	5	Medical equipment
IDWG^a^	12	A calculation formula
Blood pressure	3	Medical equipment
Blood urea nitrogen	4	Blood sample collection
Creatinine	5	Blood sample collection
Urea	2	Blood sample collection
KT/V, dialysis adequacy	5	A calculation formula
Serum albumin	14	Blood sample collection
Serum sodium	2	Blood sample collection
Serum calcium	5	Blood sample collection
Serum potassium	14	Blood sample collection
Serum phosphorus	20	Blood sample collection
Serum aluminum	1	Blood sample collection
Serum iron	1	Blood sample collection
Hemoglobin	6	Blood sample collection
Glycosylated hemoglobin	1	Blood sample collection
Hematocrit	1	Blood sample collection
Bicarbonate	1	Blood sample collection
Alkaline phosphatase	1	Blood sample collection
iPTH^b^	2	Blood sample collection
PTH^c^	4	Blood sample collection
Brain natriuretic peptide	1	Blood sample collection
C-reactive protein	1	Blood sample collection

a IDWG: intradialytic weight gain.

b iPTH: intact parathyroid hormone.

c PTH: parathyroid hormone.

## Discussion

### Principal Findings

This scoping review provides the first comprehensive analysis and summary of self-management content for patients undergoing dialysis, types of mHealth, relevant theories and models, the content of mHealth-based interventions, and methods for evaluating their effectiveness. Most studies indicate that mHealth-based interventions significantly improve self-management of patients undergoing dialysis and enhance patient outcomes [23,27,37,39,40,48].

Compared to face-to-face training, mHealth-based interventions have a greater impact on patient adherence and laboratory outcomes [67]. In this study, apps and remote monitoring systems were more common. SMS text messaging and phone calls could be used as an aid to promote self-management in patients undergoing dialysis [68]. Due to differences in mHealth-based intervention content and evaluation indicators, quantitative analysis of apps, remote monitoring systems, phones, and SMS text messaging is extremely challenging. Overall, the vast majority of apps demonstrate strong potential for improving medication adherence, enhancing care efficiency, and increasing patient satisfaction and treatment outcomes [69,70].

### Special Considerations for Specific Groups Among Patients Undergoing Dialysis

Currently, there are relatively few apps available on the market for patients undergoing dialysis. Existing research has identified 12 Android apps, 11 iOS apps, and 5 dual-platform apps closely associated with kidney disease [71]. In addition, middle-aged and older people are less receptive to apps than younger people, which is particularly reflected in lower usage rates and more negative attitudes toward apps [72]. Since middle-aged and older patients are less receptive to new things and have declining eyesight, the app should be designed to meet the special needs and usage habits of middle-aged and older patients. A survey of requirements for the app in middle-aged and older patients with CKD based on the Kano model showed that the app first had to protect privacy, followed by simplifying the data entry process, simplifying in-app navigation, and simplifying the function and number of buttons [73]. Navigation for beginners, appropriate text size, using color to distinguish different options or icons, and ensuring all buttons maintain consistency in size, labels, and spacing are also details that should be considered during software design [73]. It is also worth noting that there are also barriers that prevent the promotion of apps, such as information barriers, trustworthiness, security, compatibility, complexity, time constraints, and low mHealth literacy [68]. Therefore, in future research, user profiles can be incorporated based on patient age, dialysis modality, and digital literacy levels to automatically match interfaces [74]. During initial use, an interactive tutorial should be set up to guide patients. Beyond traditional touchscreen operations, additional features, such as voice commands, remote assistance, and offline mode, can be added to accommodate the diverse needs and usage habits of patients undergoing dialysis.

### Key Areas for Self-Management in Patients Undergoing Dialysis Using mHealth

In terms of app self-management content, CKD disease-related knowledge, symptom management, medication management, provision of health insurance information, diet management, exercise guidance, and psychosocial support may be the content they need more (arranged according to the patient’s needs) [73]. However, among the apps for patients undergoing dialysis in this study, the provision of health insurance information was what they lacked. Moreover, the app in this study focused more on disease knowledge, self-monitoring, diet, and medication management and lacked sufficient attention to exercise and psychological support. Compared to self-monitoring, dietary self-management becomes significantly more challenging for patients undergoing dialysis. Strict dietary restrictions, dynamic adjustments to meal plans (based on laboratory indicators), the need for specialized knowledge, lack of external supervision (eg, hospital and home), and the influence of long-standing dietary habits and psychological factors further compound the complexity of implementing dietary management for patients undergoing dialysis. Currently, mHealth’s promotion offers multiple solutions for dietary management among patients undergoing dialysis. The current app can automatically calculate the calories, protein, sodium, phosphorus, and potassium of foods consumed by patients undergoing dialysis, typically achieved through a nutritional database and barcode scanning functionality [25]. A study has also monitored the diet of patients undergoing dialysis by sending photos of food through an app and having them evaluated by the expert [15]. Food analysis and feedback also include the health system score, which is an easy-to-understand health score calculated by the app based on the phosphorus-to-protein ratio of the food [12]. Health care professionals provide personalized advice through dietary records and laboratory tests to promote changes in dietary behavior in patients undergoing dialysis.

Among the included studies, there were fewer studies for patients undergoing PD compared to patients undergoing hemodialysis. This may be related to the late start of PD. Self-management is an important influence on the quality of life and outcomes of patients undergoing PD [75]. Patients undergoing PD who undergo inappropriate operations face a higher risk of developing peritonitis [76]. If patients undergoing PD experience a technical failure, they must be treated with hemodialysis. Compared with PD, long-term regular hemodialysis significantly impacts patients’ quality of life, hinders their social reintegration, and increases the risk of complications [77,78]. Therefore, for patients undergoing PD, self-management is necessary for them to master. The focus of self-management in patients undergoing PD is not the same as in patients undergoing hemodialysis. As patients undergoing PD need to perform peritoneal dialysis-related operations at home, training in relevant knowledge and operational skills is particularly important [76]. The training of skills mainly includes aseptic operation, change of peritoneal fluid and peritoneal dialysis catheter, and outlet care [79]. Patients undergoing PD also need to learn the calculation of ultrafiltration [79]. All of these can be learned and managed remotely based on mHealth. In the event of an emergency (eg, contamination or disconnection of the peritoneal dialysis tubing), patients should immediately contact a health care professional for on-site guidance. Thus, establishing emergency contact channels within the app is particularly crucial.

### Theoretical or Model Support for mHealth Intervention Programs in Patients Undergoing Dialysis

Many theories and models have been used to guide practice in the self-management of patients undergoing dialysis. TTM, self-efficacy theory, and social cognitive theory are the most commonly used theories [12,20,40,42]. Currently, most studies use a single theory as their guiding framework, with fewer adopting 2 or more theories for guidance. Taking the TTM as an example, it emphasizes guiding patients through staged behavioral shifts but overlooks individual differences (such as cultural background and cognitive level) [20,22,24,26,42]. Self-care theory, on the other hand, emphasizes personalized care needs and can be combined with TTM. Additionally, the maintenance phase of TTM is prone to behavioral relapse, particularly among patients requiring lifelong treatment (eg, dialysis). A single theory cannot sustainably motivate patients. Integrating other theories (such as the PERMA [Positive Emotion, Engagement, Relationship, Meaning and Accomplishment] model [80]) during this phase can further reinforce patients’ behavior. Therefore, future research should focus more on integrating multiple theories rather than relying on a single one. In addition, goal-setting theory, the information-motivation-behavioral skills model, the chronic care model, the ecological model of health behavior, and the theory of planned behavior can also be applied in subsequent research.

### Implications for Future Research

Future research on self-management software for patients undergoing dialysis will focus on integrating the strengths of existing tools and promoting their synergistic use. Software development should break down barriers between tools, such as deeply integrating the real-time data collection capabilities of remote patient monitoring systems, the personalized analysis functions of intelligent decision support systems, and the instant communication advantages of phone or SMS text messaging, to provide patients with more comprehensive self-management support.

A combination of artificial intelligence can be considered for use in the remote management of patients undergoing dialysis (eg, wearable devices). Smart wristbands can monitor data, such as heart rate, blood pressure, activity, and sleep [81] and automatically transmit the data to the app for analysis and storage. Some researchers have implemented data linkage through apps using near field communication and optical character recognition. Data from measuring devices, such as sphygmomanometers and weight scales, can be automatically transmitted to the app via near field communication [14]. Alternatively, the numbers from the sphygmomanometer can be captured using the phone’s camera and imported into the app [14]. These not only improve the efficiency of patients undergoing dialysis but also increase the accuracy of the records. In conclusion, there is still a lot of room to explore the use of artificial intelligence in combination with mHealth in patients undergoing dialysis.

Different studies have different insights into the evaluation criteria for self-management. Most of the studies used objective indicators as one of the evaluation criteria for self-management. For the evaluation of knowledge of patients undergoing dialysis, most studies have assessed it using self-developed questionnaires and lacked uniform criteria for judging. Various scales are currently available for assessing self-management, adherence, self-efficacy, and quality of life. The use of these assessment tools varies across different studies. Existing research lacks a gold standard for evaluating self-management in patients undergoing dialysis. Therefore, there is a need to standardize the criteria for evaluating self-management in patients undergoing dialysis in future studies.

This study provides guidance for the development of subsequent dialysis-related software for patients, including the design of functional modules, user experience optimization, and the integration of clinical indicators and assessment tools. Our findings will enhance the ability of patients undergoing dialysis to self-manage their health at home, improving both the effectiveness of their dialysis treatment and their quality of life. In the future design of the app, attention should also be paid to the usage needs of special groups, strengthening adaptive software development, deeply integrating artificial intelligence technology, and establishing standardized self-management evaluation criteria.

### Limitation

This scoping review also has some limitations. First, the broad scope of scoping reviews and the complexity of search strategies may lead to the omission of relevant studies. These challenges persist despite strict adherence to the PRISMA-ScR guidelines for greater rigor and transparency. Second, self-management assessment tools incorporate numerous subjective and objective indicators, which complicate data integration and comparability. Third, publication bias was one of the limitations of this study. This review only covered relevant studies published in English. Most of the included studies were limited to Asia and North America, which may have resulted in limited global generalizability. Future studies should use standardized, integrated measures to improve consistency and reliability. Additionally, these shortcomings can be remedied by improving search strategies, expanding database coverage, and removing language restrictions to include a more diverse patient population from different continents.

### Conclusion

This study conducted a scoping review of the existing literature on mHealth-based self-management among patients undergoing dialysis. Although mHealth holds potential advantages for self-management in patients undergoing dialysis, it has not been widely adopted and integrated into standard renal care, requiring further optimization and refinement. This study provides theoretical and practical guidance for subsequent research, helping to enhance self-management of patients undergoing dialysis and improve their quality of life, ultimately offering insights for digital transformation in chronic disease management.

10.2196/76880 Multimedia Appendix 1 Search strategy

10.2196/76880 Multimedia Appendix 2 Critical appraisal of the selected studies using the Mixed Methods Appraisal Tool (MMAT).

10.2196/76880 Multimedia Appendix 3 Types of mobile health, core intervention content, intervention time, and evaluation indicators for self-management in patients undergoing dialysis.

10.2196/76880 Checklist 1 PRISMA-ScR and PRISMA-S checklists.

## Abbreviations

CKD: chronic kidney diseases
ESRD: end-stage renal disease
mHealth: mobile health
PD: peritoneal dialysis
PERMA: Positive Emotion, Engagement, Relationship, Meaning and Accomplishment
PRISMA-S: Preferred Reporting Items for Systematic Reviews and Meta-Analyses Literature Search Extension
PRISMA-ScR: Preferred Reporting Items for Systematic Reviews and Meta-Analyses Extension for Scoping Reviews
TTM: transtheoretical model
