# Supplementary material for: The Application of Mobile Health in Self-Management Among Patients Undergoing Dialysis: Scoping Review
Source: J Med Internet Res. 2026 Jan 2;28:e76880. doi: 10.2196/76880 (PMC12791203; doi:10.2196/76880)
Supplement: Multimedia Appendix 1 [file jmir-v28-e76880-s001.docx]

**Search strategy**

This study updated all initial databases by re-executing the original search strategy. During the update, the exact same keyword combinations and filtering criteria (language, publication date) used in the initial search were strictly applied, with only the search cutoff date extended to October 20, 2025.

**Pubmed (n=478)**

#1 (((((((((((((((("chronic kidney disease") OR ("CKD")) OR (dialysis)) OR ("haemodialys*")) OR ("hemodialys*")) OR ("chronic kidney failure")) OR ("renal dialysis")) OR ("home hemodialysis")) OR ("home haemodialysis")) OR ("peritoneal dialysis")) OR (hemodiafiltration)) OR (hemoperfusion)) OR ("ESRD")) OR ("end stage renal disease")) OR ("renal replacement therapy")) OR ("kidney transplant*")) OR ("renal transplant*")

#2 (((((((((((((((((((((((((((mHealth) OR ("m health")) OR (eHealth)) OR ("e Health")) OR ("digital health")) OR ("mobile health")) OR ("mobile technology")) OR ("personal digital assistant*")) OR ("portable electronic application*")) OR ("portable software application*")) OR ("remote monitoring")) OR ("remote patient monitoring")) OR ("remote patient management")) OR ("remote biometric monitoring")) OR ("tablet* computer*")) OR ("phone app*")) OR ("cellphone app*")) OR ("telephone app*")) OR ("mobile app*")) OR ("mobile phone*")) OR ("smart phone*")) OR ("cellular phone*")) OR ("cell phone*")) OR ("telenephro*")) OR (wearable)) OR ("text messages")) OR ("telecommunication*")) OR (sensor)

#3 ("Self Care"[Mesh]) OR ((((((((((((("care, self") OR ("self management")) OR ("disease management")) OR ("patient-centred care")) OR (patient centred care)) OR ("patient centered")) OR ("self monitor*")) OR ("self efficacy")) OR ("self regulat*")) OR (patient orient* monitor*) OR (patient-orient* monitor*) OR ("health educat*")) OR ("health promot*")) OR ("patient educat*")) OR ("health behavior*"))

#4 ("2010/01/01"[Date - Publication] : "2025/10/20"[Date - Publication])

#5 #1 AND #2 AND #3 AND #4

**CINAHL (n=171)**

#1 ("chronic kidney disease" OR "CKD" OR dialysis OR "haemodialys*" OR "hemodialys*" OR "chronic kidney failure" OR "renal dialysis" OR "home hemodialysis" OR "home haemodialysis" OR "peritoneal dialysis" OR hemodiafiltration OR hemoperfusion OR "ESRD" OR "end stage renal disease" OR "renal replacement therapy" OR "kidney transplant*" OR "renal transplant*")

#2 ("mHealth" OR "m health" OR "eHealth" OR "e Health" OR "digital health" OR "mobile health" OR "mobile technology" OR "personal digital assistant*" OR "portable electronic application*" OR "portable software application*" OR "remote monitoring" OR "remote patient monitoring" OR "remote patient management" OR "remote biometric monitoring" OR "tablet* computer*" OR ("phone app" OR "phone application" OR "phone applications" OR "phone apps") OR "cellphone app*" OR ("telephone applications") OR ("mobile app" OR "mobile application" OR "mobile applications" OR "mobile apps") OR ("mobile phone" OR "mobile phones") OR ("smart phone" OR "smart phones") OR ("cellular phone" OR "cellular phones") OR ("cell phone") OR "telenephro*" OR wearable OR "text messages" OR "telecommunication*" OR "sensor")

#3 ("Self Care" OR "care, self" OR "self management" OR "disease management" OR "patient-centred care" OR "patient centred care" OR "patient centered" OR "self monitor*" OR "self efficacy" OR "self regulat*" OR "patient orient* monitor*" OR "patient-orient* monitor*" OR "health educat*" OR "health promot*" OR "patient educat*" OR "health behavior*")

#4 #1 AND #2 AND #3

#5 Qualification condition- Published date: 2010/01/01 - 2025/10/20

**PsychlNFO (n=67)**

#1 "chronic kidney disease" or "CKD" or dialysis or "haemodialys*" or "hemodialys*" or "chronic kidney failure" or "renal dialysis" or "home hemodialysis" or "home haemodialysis" or "peritoneal dialysis" or hemodiafiltration or hemoperfusion or "ESRD" or "end stage renal disease" or "renal replacement therapy" or "kidney transplant*" or "renal transplant*"
dialysis or "haemodialys*" or "hemodialys*" or "chronic kidney failure" or "renal dialysis" or "home hemodialysis" or "home haemodialysis" or "peritoneal dialysis" or hemodiafiltration or hemoperfusion or "renal replacement therapy"

#2 mHealth OR 'm health' OR eHealth OR 'e Health' OR 'digital health' OR 'mobile health' OR 'mobile technology' OR 'personal digital assistant*' OR 'portable electronic application*' OR 'portable software application*' OR 'remote monitoring' OR 'remote patient monitoring' OR 'remote patient management' OR 'remote biometric monitoring' OR 'tablet* computer*' OR ('phone app' OR 'phone application' OR 'phone applications' OR 'phone apps') OR 'cellphone app*' OR ('telephone applications') OR ('mobile app' OR 'mobile application' OR 'mobile applications' OR 'mobile apps') OR ('mobile phone' OR 'mobile phones') OR ('smart phone' OR 'smart phones') OR ('cellular phone' OR 'cellular phones') OR ('cell phone') OR 'telenephro*' OR wearable OR 'text messages' OR 'telecommunication*' OR sensor

#3 ('Self Care' OR 'care, self' OR 'self management' OR 'disease management' OR 'patient-centred care' OR 'patient centred care' OR 'patient centered' OR 'self monitor*' OR 'self efficacy' OR 'self regulat*' OR 'patient orient* monitor*' OR 'patient-orient* monitor*' OR 'health educat*' OR 'health promot*' OR 'patient educat*' OR 'health behavior*')

#4 #1 AND #2 AND #3

#5 Qualification condition- Published date: 2010/01/01 - 2025/10/20

**Embase (n=251)**

#1 ('chronic kidney disease' or 'CKD' or dialysis or 'haemodialys*' or 'hemodialys*' or 'chronic kidney failure' or 'renal dialysis' or 'home hemodialysis' or 'home haemodialysis' or 'peritoneal dialysis' or hemodiafiltration or hemoperfusion or 'ESRD' or 'end stage renal disease' or 'renal replacement therapy' or 'kidney transplant*' or 'renal transplant*').mp. [mp=title, abstract, heading word, drug trade name, original title, device manufacturer, drug manufacturer, device trade name, keyword heading word, floating subheading word, candidate term word]

#2 (mHealth OR 'm health' OR eHealth OR 'e Health' OR 'digital health' OR 'mobile health' OR 'mobile technology' OR 'personal digital assistant*' OR 'portable electronic application*' OR 'portable software application*' OR 'remote monitoring' OR 'remote patient monitoring' OR 'remote patient management' OR 'remote biometric monitoring' OR 'tablet* computer*' OR ('phone app' OR 'phone application' OR 'phone applications' OR 'phone apps') OR 'cellphone app*' OR ('telephone applications') OR ('mobile app' OR 'mobile application' OR 'mobile applications' OR 'mobile apps') OR ('mobile phone' OR 'mobile phones') OR ('smart phone' OR 'smart phones') OR ('cellular phone' OR 'cellular phones') OR ('cell phone') OR 'telenephro*' OR wearable OR 'text messages' OR 'telecommunication*' OR sensor).mp. [mp=title, abstract, heading word, drug trade name, original title, device manufacturer, drug manufacturer, device trade name, keyword heading word, floating subheading word, candidate term word]

#3 ('Self Care' OR 'care, self' OR 'self management' OR 'disease management' OR 'patient-centred care' OR 'patient centred care' OR 'patient centered' OR 'self monitor*' OR 'self efficacy' OR 'self regulat*' OR 'patient orient* monitor*' OR 'patient-orient* monitor*' OR 'health educat*' OR 'health promot*' OR 'patient educat*' OR 'health behavior*').mp. [mp=title, abstract, heading word, drug trade name, original title, device manufacturer, drug manufacturer, device trade name, keyword heading word, floating subheading word, candidate term word]

#4 #1 AND #2 AND #3

#5 limit 4 to yr="2010 -Current"

**Web of Science (n=516)**

#1 TS=("chronic kidney disease" or "CKD" or dialysis or "haemodialys*" or "hemodialys*" or "chronic kidney failure" or "renal dialysis" or "home hemodialysis" or "home haemodialysis" or "peritoneal dialysis" or hemodiafiltration or hemoperfusion or "ESRD" or "end stage renal disease" or "renal replacement therapy" or "kidney transplant*" or "renal transplant*") and Preprint Citation Index (Exclude – Database)

#2 TS=(mealth or "m health" or eHealth or "e Health" or "digital health" or "mobile health" or "mobile technology" or "personal digital assistant*" or "portable electronic application*" or "portable software application*" or "remote monitoring" or "remote patient monitoring" or "remote patient management" or "remote biometric monitoring" or "tablet* computer*" or "phone app*" or "cellphone app*" or "telephone app*" or "mobile app*" or "mobile phone*" or "smart phone*" or "cellular phone*" or "cell phone*" or "telenephro*" or wearable or "text messages" or "telecommunication*" or sensor) and Preprint Citation Index (Exclude – Database)

#3 TS=("Self Care" or "care, self" or "self management" or "disease management" or "patient-centred care" or "patient centred care" or "patient centered" or "self monitor*" or "self efficacy" or "self regulat*" or "patient orient* monitor*" or "patient-orient* monitor*" or "health educat*" or "health promot*" or "patient educat*" or "health behavior*") and Preprint Citation Index (Exclude – Database)

#4 #1 AND #2 AND #3

#5 Qualification condition- Published date: 2010/01/01 - 2025/10/20
