# Supplementary material for: The Application of Mobile Health in Self-Management Among Patients Undergoing Dialysis: Scoping Review
Source: J Med Internet Res. 2026 Jan 2;28:e76880. doi: 10.2196/76880 (PMC12791203; doi:10.2196/76880)
Supplement: Multimedia Appendix 3 [file jmir-v28-e76880-s003.docx]

Table S1 Types of mHealth, core intervention content, intervention time and evaluation indicators for self-management in patients undergoing dialysis patients.

| Author, year | Country | Population | mHealth category | Main elements of self-management | Intervention time | Evaluation Content |
| --- | --- | --- | --- | --- | --- | --- |
| Saadatifar etc, 2022  [36] | Iran | HD | Dialysis-integrated software and instant messaging software | (1) Disease-related knowledge;  (2) Self-monitoring of disease;  (3) Diet and fluid management;  (4) Exercise management;  (5) Medication management. | 3 months | ● Scales: treatment adherence. |
| Ren etc, 2019[34] | China | HD | Instant messaging software | (1) Disease-related knowledge;  (2) Psychological management. | 3 months | ● Scales: self-management, self-efficacy, and knowledge. |
| Park etc, 2019[31] | Korea | HD | Dialysis-integrated software, SMS | (1) Disease-related knowledge;  (2) Self-monitoring of disease;  (3) Diet and fluid management;  (4) Medication management. | 8 weeks | ● Scales: treatment adherence, self-efficacy;  ● Laboratory indicators: serum potassium and phosphorus;  ● Dry weight, IDWG. |
| Pack etc, 2021[30] | Korea | HD | Dialysis-integrated software | (1) Diet and fluid management. | 8 weeks | ● Scales: self-efficacy, quality of life;  ● Laboratory indicators: serum phosphorus, potassium and albumin. |
| Khoury etc, 2020[27] | Netherlands | HD | Dialysis-integrated software | (1) Diet and fluid management. | 2 weeks | ● Scales: diet and nutrition;  ● Laboratory indicators: serum phosphorus, potassium, iron, albumin, aluminum. |
| Nasrin etc, 2019[24] | Iran | HD | Telephones | (1) Diet and fluid management;  (2) Medication management. | 12 weeks | ● Laboratory indicators: urea nitrogen, serum sodium, potassium, calcium, phosphorus, albumin;  ● IDWG. |
| Cho etc, 2020[20] | Korea | HD | Tablet-based video management project | (1) Disease-related knowledge. | 6 weeks | ● Scales: knowledge, anxiety, self-care behavior;  ● Laboratory indicators: serum potassium, phosphorus, albumin, KT/V;  ● IDWG. |
| Chiang etc, 2021[12] | Taiwan, China | HD | Dialysis-integrated software | (1) Diet and fluid management. | 3 months | ● Scales: knowledge, self-efficacy;  ● Laboratory indicators: serum phosphorus, albumin. |
| Zwi etc, 2022[41] | Australia | HD | Dialysis-integrated software | (1) Disease-related knowledge. | 12 weeks | ● Scales: knowledge and confidence, health behaviour, self-efficacy, health literacy, quality of life. |
| Welch etc, 2013[40] | USA | HD | Dialysis-integrated software | (1) Diet and fluid management. | 6 weeks | ● Scales: self-efficacy, perceived benefits and perceived control, diet and nutrition;  ● IDWG. |
| Thongsunti etc, 2024  [39] | Thailand | HD | Instant messaging software | (1) Disease-related knowledge;  (2) Diet and fluid management;  (3) Medication management. | 24 weeks | ● Scales: knowledge, self-efficacy, diet and nutrition, treatment adherence;  ● Laboratory indicators: serum phosphorus. |
| Chung etc, 2024[21] | Korea | HD | SMS | (1) Disease-related knowledge;  (2) Diet management (home-delivered meals). | 8 weeks | ● Scales: self-efficacy, depression, diet and nutrition, treatment adherence, quality of life;  ● Laboratory indicators: serum phosphorus, potassium. |
| Dawson etc, 2021[22] | Australia | HD | SMS | (1) Diet and fluid management. | 6 months | ● Scales: diet and nutrition, diet adherence, quality of life;  ● Laboratory indicators: serum potassium, phosphorus, bicarbonate, urea, albumin, parathyroid hormone, glycosylated hemoglobin;  ● IDWG. |
| Khoury etc, 2021[23] | Netherlands | HD | Dialysis-integrated software | (1) Diet and fluid management. | 2 weeks | ● Scales: knowledge, diet and nutrition, diet and fluid adherence;  ● Laboratory indicators: serum phosphorus. |
| Hosseini etc, 2023[26] | Iran | HD | Dialysis-integrated software | (1) Disease-related knowledge;  (2) Self-monitoring of disease;  (3) Diet and fluid management;  (4) Exercise management;  (5) Medication management. | 6 months | ● Scales: self-efficacy, self-care. |
| Min etc, 2020[13] | Korea | HD | Dialysis-integrated software | (1) Disease-related knowledge;  (2) Self-monitoring of disease;  (3) Diet and fluid management;  (4) Medication management. | 10 weeks | ● Scales: behavioral compliance, psychology, self-efficacy;  ● Laboratory indicators: serum phosphorus, albumin;  ● IDWG. |
| Mollaoğlu etc, 2024  [28] | Turkey | HD | Instant messaging software | (1) Disease-related knowledge;  (2) Diet and fluid management;  (3) Psychological management. | 12 weeks | ● Scales: behavioral compliance, psychology;  ● Laboratory indicators: urea nitrogen, creatinine;  ● Weight, dry weight, IDWG. |
| Rocco etc, 2023[35] | USA | HD | Dialysis-integrated software | (1) Fluid management. | 4 weeks | ● IDWG. |
| David etc, 2021[37] | USA | HD | Dialysis-integrated software and email | (1) Disease-related knowledge;  (2) Diet and fluid management;  (3) Medication management. | 6 months | ● Scales: diet and nutrition, adherence;  ● Laboratory indicators: serum albumin and phosphorus. |
| Teong etc, 2022[38] | Malaysia | HD | Dialysis-integrated software | (1) Disease-related knowledge;  (2) Diet and fluid management;  (3) Medication management. | 12 weeks | ● Scales: diet and nutrition, adherence, knowledge;  ● Laboratory indicators: serum phosphorus, calcium, intact parathyroid hormone, alkaline phosphatase, and albumin. |
| Pungchompoo etc, 2024[33] | Thailand | HD | Dialysis-integrated software and phone | (1) Disease-related knowledge;  (2) Self-monitoring of disease;  (3) Psychological management. | 6 months | ● Scales: quality of life;  ● Laboratory indicators: urea nitrogen, creatinine, hemoglobin, hematocrit, albumin, potassium, KT/V, and standardized proteolysis rate. |
| Nursalam etc,  2020[29] | Indonesia | HD | Dialysis-integrated software | (1) Disease-related knowledge;  (2) Self-monitoring of disease;  (3) Diet and fluid management. | Not mentioned | ● Scales: adherence, self-efficacy, perceived benefit, perceived threat and perceived barrier;  ● IDWG. |
| Hayashil etc,  2017[25] | Japan | HD | A remote patient monitoring system | (1) Self-monitoring of disease. | 2 weeks | ● Scales: quality of life;  ● Laboratory indicators: serum potassium, phosphorus;  ● IDWG. |
| Pinto etc, 2020[32] | Brazil | HD | Dialysis-integrated software | (1) Diet and fluid management. | 3 months | ● Scales: self-management, quality of life;  ● Laboratory indicators: serum potassium, phosphorus, calcium, parathyroid hormone, KT/V;  ● IDWG. |
| Andriati etc,  2025[42] | Indonesia | HD | Dialysis-integrated software | (1) Disease-related knowledge;  (2) Diet and fluid management;  (3) Medication management;  (4) Psychological management. | 3 months | ● Scales: adherence;  ● Laboratory indicators: urea, creatinine. |
| Taguiam etc,  2025[43] | USA | HD | Dialysis-integrated software | (1) Disease-related knowledge;  (2) Self-monitoring of disease;  (3) Diet and fluid management;  (4) Medication management. | 4 weeks | ● Scales: adherence;  ● IDWG. |
| Lee etc, 2024[15] | Korea | PD | Instant messaging software | (1) Diet and fluid management. | 5 weeks | ● Scales: self-efficacy, adherence;  ● Laboratory indicators: serum potassium, phosphorus, albumin, hemoglobin. |
| Chae etc, 2024[46] | Korea | PD | Dialysis-integrated software, phone, SMS | (1) Disease-related knowledge;  (2) Self-monitoring of disease;  (3) Diet and fluid management;  (4) Medication management. | 10 weeks | ● Scales: knowledge, self-efficacy, adherence, quality of life;  ● Laboratory indicators: serum potassium, phosphorus, albumin, hemoglobin. |
| Uchiyama etc, 2022[44] | Japan | PD | A remote patient monitoring system | (1) Self-monitoring of disease. | 24 weeks | ● Scales: quality of life;  ● Laboratory indicators: serum parathyroid hormone, urea nitrogen, creatinine, sodium, potassium, albumin, calcium, phosphorus, hemoglobin, C-reactive protein, brain natriuretic peptide;  ● PD-related indicators: KT/V, ultrafiltration, creatinine clearance;  ● Blood pressure. |
| Jung etc, 2021[45] | Korea | PD | A remote patient monitoring system | (1) Self-monitoring of disease. | 13 weeks | ● Scales: quality of life;  ● PD-related indicators: dialysis adherence, accuracy, overhydration, adequacy (KT/V);  ● Blood pressure. |
| Eakalak etc, 2022[14] | Thailand | PD | Dialysis-integrated software | (1) Self-monitoring of disease. | 20 weeks | ● Completion rate, clinic contact frequency. |
| Zeng etc, 2025[47] | China | PD | Dialysis-integrated software | (1) Disease-related knowledge;  (2) Self-monitoring of disease;  (3) Diet and fluid management;  (4) Medication management. | Not applicable | ● Scales: adherence, quality of life;  ● Laboratory indicators: serum intact parathyroid hormone, potassium, albumin, calcium, phosphorus, hemoglobin;  ● Blood pressure. |
| Stark etc, 2011[48] | USA | PD, HD | Dialysis-integrated software, phone | (1) Diet and fluid management. | 16 weeks | ● Dialysis adherence-completion rate. |
| Beer etc, 2025[49] | Australia | PD, HD | Dialysis-integrated software | (1) Disease-related knowledge;  (2) Self-monitoring of disease;  (3) Diet and fluid management;  (4) Medication management. | 3 months | ● Scales: self-management, adherence, quality of life;  ● Laboratory indicators: serum phosphorus. |

Abbreviations: Hemodialysis, HD; peritoneal dialysis, PD; application, APP; short message service, SMS; intradialytic weight gain, IDWG.

Table S2 Frequency distribution of mHealth-based self-management intervention times for patients undergoing dialysis patients.

| Intervention time | Frequency |
| --- | --- |
| 3 months | 6 |
| 6 months | 4 |
| 2 weeks | 3 |
| 4 weeks | 2 |
| 5 weeks | 1 |
| 6 weeks | 2 |
| 8 weeks | 3 |
| 10 weeks | 2 |
| 12 weeks | 4 |
| 13 weeks | 1 |
| 16 weeks | 1 |
| 20 weeks | 1 |
| 24 weeks | 2 |
